# Research progress on antidepressant effects and mechanisms of berberine

**DOI:** 10.3389/fphar.2024.1331440

**Published:** 2024-01-22

**Authors:** Yang Gao, Kexin Nie, Hongzhan Wang, Hui Dong, Yueheng Tang

**Affiliations:** ^1^ Institute of Integrated Traditional Chinese and Western Medicine, Tongji Hospital, Tongji Medical College, Huazhong University of Science and Technology, Wuhan, Hubei, China; ^2^ Department of Rehabilitation Medicine, Tongji Hospital, Tongji Medical College, Huazhong University of Science and Technology, Wuhan, Hubei, China

**Keywords:** berberine, depression, neurotransmitter, cell regeneration, HPA axis, oxidative stress, inflammation

## Abstract

Depression, a global health problem with growing prevalence, brings serious impacts on the daily life of patients. However, the antidepressants currently used in clinical are not perfectly effective, which greatly reduces the compliance of patients. Berberine is a natural quaternary alkaloid which has been shown to have a variety of pharmacological effects, such as hypoglycemic, lipid-regulation, anti-cancer, antibacterial, anti-oxidation, anti-inflammatory, and antidepressant. This review summarizes the evidence of pharmacological applications of berberine in treating depression and elucidates the mechanisms of berberine regulating neurotransmitter levels, promoting the regeneration of hippocampal neurons, improving hypothalamic-pituitary-adrenal axis dysfunction, anti-oxidative stress, and suppressing inflammatory status in order to provide a reference for further research and clinical application of berberine.

## 1 Introduction

Depression is a common mood disorder characterized by low mood, anxiety, insomnia, loss of appetite, and poor concentration (Malhi and Mann, 2018; McCarron et al., 2021). In 2021, approximately 280 million people suffered from depression with a global prevalence rate of 3.8%, and severe depression can even lead to suicide, with more than 700,000 deaths due to suicide each year (World Health Organization, 2022). Meanwhile, depression is the sixth leading cause of disability-adjusted life-years (DALYs) among 20- to 50-year-olds (GBD, 2019 Diseases and Injuries Collaborators, 2020). As a result, depression significantly diminishes the quality of life and places a huge burden on the global economy (Chisholm et al., 2016). However, depression is frequently comorbid with other mental and chronic medical conditions (Berk et al., 2023). Clinical diagnosis of depression relies on the identification of several key symptoms mentioned above, while it is difficult to diagnose due to none of the symptoms is pathognomonic for depression (Malhi and Mann, 2018). Therefore, it is critical to improve early detection and management for people suffering from depression due to the complexity and seriousness of the pathophysiology of depression.

Selective serotonin reuptake inhibitors (SSRIs), serotonin and norepinephrine reuptake inhibitors (SNRIs), monoamine oxidase inhibitors (MAOIs), and tricyclic and tetracyclic antidepressants are the most commonly used antidepressants in clinical, with small differences between them were found (Cipriani et al., 2018). Most of these drugs are slow-acting, one-third of patients with major depressive disorder (MDD) did not improve significantly after taking multiple consecutive courses of antidepressants (Jha and Mathew, 2023). A significant proportion of patients develop treatment-resistant depression, requiring medication changes, additional treatment cycles, or adjunctive therapies (Lundberg et al., 2023). Besides, most of these medications have side effects including but not limited to gastrointestinal reactions, hepatotoxicity and hypersensitivity reactions, weight gain and metabolic disturbances, sexual dysfunction, and sleep disturbances (Gill et al., 2020; Rothmore, 2020; Oliva et al., 2021). Carvalho et al. demonstrated that the long-term treatment with these novel-generation antidepressant drugs should be avoided if alternative treatments are available (Carvalho et al., 2016). It is evident that the current treatment of depression remains difficult and there is an urgent need to find safer and more effective antidepressant drugs.

Traditional Chinese medicine (TCM) profits from the flexible utilization of the concepts of holistic view and diagnosis and treatment in clinical, plays a role in the treatment of multiple diseases, especially in the fight against COVID-19, which has shown the world the great potential of TCM (Lyu et al., 2021; Chen et al., 2022). In recent years, the antidepressant effects of herbal formulas and individual components have been gradually confirmed (Li et al., 2020; He et al., 2022). For instance, Lu et al. found that the methanol extract, ethanol extract, aqueous extract, and its volatile oil of *Rhizoma Cyperi* have antidepressant activity (Lu et al., 2022); muscone may alleviate lipopolysaccharide (LPS)-induced depression-like behaviors through TLR/MyD88 and TLR4/NLRP3 pathways (He et al., 2020); Lily Bulb and Rehmannia decoction can improve depression by reducing MAO activity, increasing monoamine neurotransmitter levels and regulating the hypothalamic-pituitary-adrenal (HPA) axis dysfunction (Chi et al., 2019; Zhang et al., 2021), a meta-analysis showed that chaihu-jia-longgu-muli-tang can ameliorate the depressive manifestations of the patients via suppressing inflammation (Zhao et al., 2023).

**FIGURE 1:**
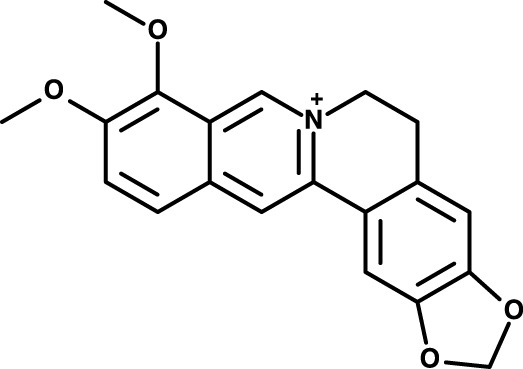
Chemical structure berberine.

Berberine (Ber, Figure 1), a quaternary ammonium alkaloid, is one of the important constituents of Chinese herbal medicines such as *Rhizoma Coptidis*, *Rhizoma Cyperus*, and *Rhizoma Rhei*. Studies have shown that Ber possesses a variety of biological activities (Song et al., 2020), including hypoglycemic (Xie et al., 2022), hypolipidemic (Wang et al., 2022), antimicrobial (Jamshaid et al., 2020), anti-inflammatory (Li et al., 2020), and antitumor (Liu et al., 2020), etc. Therefore, the exploration of the pharmacological effects of Ber and its derivatives has a broad prospect. In recent years, the role of Ber in the neuropsychiatric field has greatly attracted the attention of researchers, and a large number of studies have been conducted to explore the effects of Ber on neuropsychiatric diseases including anxiety disorders and Alzheimer’s disease (Akbar et al., 2021; Fang et al., 2021; Raju et al., 2021; YU et al., 2021; Nguyen et al., 2022).

Studies have shown that multiple factors are involved in the pathogenesis of depression, such as low functioning of the monoamine nervous system (Borroto-Escuela et al., 2021), inflammation (Beurel et al., 2020; Novakovic et al., 2023), nerve damage and regeneration disorders (Tartt et al., 2022; Thompson, 2023), dysfunction of the HPA axis (Herzog et al., 2023), oxidative stress (Bhatt et al., 2020), and genetic and psychosocial factors (Zhao et al., 2022; Ross et al., 2023), etc. The pathogenesis fits right in with the broad pharmacologic effects of Ber as mentioned above, and an increasing number of *in vitro* and *in vivo* experiments have focused on the validation of the antidepressant effects of Ber (Zhu et al., 2017; LU et al., 2021; YU et al., 2021; Wang et al., 2022b). Therefore, in this paper, we searched for related studies in databases such as PubMed, ScienceDirect, Web of Science, Chinese National Knowledge Infrastructure (CNKI), and Wanfang Data Resource System Chinese Science databases from inception till October 2023 to review the potential mechanisms of Ber in the treatment of depression, in order to provide a scientific basis for its in-depth study and clinical application. Table 1 presents relevant information on the studies focused on the antidepressant effects of Ber.

**TABLE 1:** The *in vivo* studies on the antidepressant effects of berberine.

Ref.	Animals	Modeling methods	Drugs	Usage	Dose and duration	Evaluation methods	Functions
Zhang et al. (2021)	SD rats	CUMS	Ber plus GRb1	i.g	150 mg/kg Ber and 20 mg/kg GRb1, for 4 weeks	FST, SPT, EPM, OFT	Ber + GRb1 reduced the immobility time in FST, upregulated the percentages of sugar water preference in SPT and the activity times in EPM, and increased the maximum travel distance, total travel distance, and time spent at the area center in the OFT.
Xu et al. (2021)	SD rats	reserpine intervention	Ber plus EVO	i.n	0.05, 0.10, 0.15 mg/kg Ber and 0.008, 0.017, 0.025 mg/kg EVO, not mentioned	eyelid ptosis score	Ber plus EVO increased eyelid ptosis score
Zhu et al. (2017)	SD rats	CUMS	Ber	not mentioned	40, 200 mg/kg, not mentioned	OFT, FST, SPT	Ber (200 mg/kg) increased the traversing times, vertical movement, and grooming times in the OFT, reduced the motionless time in the FST, and improved the sucrose preference in the SPT.
Lee et al. (2012)	SD rats	morphine withdrawal	Ber	i.p	10, 20, and 50 mg/kg, for 10 days	FST, EPM	Ber (50 mg/kg) decreased the immobility time and restored climbing behavior in the FST, increased the time in the open arms, and the number of entries into the open arms of the maze in the EPM.
Huang et al. (2023)	Wistar rats	CUMS	Ber	i.g	50, 100 mg/kg, for 14 days	OFT, SPT	Ber increased the rearing numbers and total distances in the OFT and prevented CUMS-induced abnormal SPT.
Wang et al. (2020)	Wistar rats	CUMS	BBH/HP-β-CD inclusion complex	i.n	0.05, 0.10, and 0.15 mg/kg, for 14 days	OFT, SPT	BBH/HP-β-CD inclusion complex increased the number of rearing and total distance in the OFT, and improved the sucrose intake in the SPT.
Wang et al. (2022)	ICR mice	CUMS	Berberine	p.o	25, 50, and 100 mg/kg, for 21 days	SPT, FST, TST, OFT, NSFT	Ber increased the sucrose preference in the SPT, improved the immobility time in the FST and TST, increased the number of crossings, rearing, and total moving distance in the OFT, and decreased the latency to feed in the NSFT.
Fan et al. (2017)	ICR mice	ovariectomize	Ber	i.p	5, 10 mg/kg, for 7 days	OFT, FST	Ber (10 mg/kg) reduced the immobility time in the FST, and did not affect locomotor activity or rearing in the OFT.
Liu et al. (2017)	ICR mice	CUMS	Ber	p.o	50, 100 mg/kg, for 4 weeks	SPT, NSFT	Ber increased the sucrose preference in the SPT, 100 mg/kg Ber decreased the latency to feed in the NSFT.
Shen et al. (2016)	ICR mice	CORT intervention	Ber	i.g	50, 100 mg/kg, for 21 days	SPT, FST, OFT	Ber increased the sucrose preference in the SPT and the immobility time in the FST, while having no significant effects on the number of crossings and rearing in the OFT.
Qin et al. (2023)	C57BL/6N mice	CORT intervention	Ber	i.g	100, 200 mg/kg, for 28 days	OFT, TST, FST, SPT	Ber (200 mg/kg increased the center zone time duration, total distance as well as zone transition number in OFT, reduced immobile duration in the FST and TST, and improved the consumption of sucrose solution in SPT.
Yang et al. (2023)	C57BL/6J mice	CUMS	Ber	i.g	5, 10 mg/kg, for 3 weeks	SPT, FST, TST, OFT	Ber increased the proportion of sucrose preference in SPT and the immobility in the TST and FST, the difference in the distance traveled in the OFT was little
Zhan et al. (2021)	C57BL/6J mice	CUMS	Ber	i.g	20 mg/kg, for 14 days	FST, SPT	Ber improved the sucrose preference in the SPT and increased the swimming time in the FST.
Yi et al. (2021)	C57BL/6J mice	CUMS	Ber	i.g	100 mg/kg, for 7 days	SPT, NSFT	Ber increased the sucrose preference in the SPT and reduced the latency to feed in the NSFT.
Ge et al. (2023)	C57BL/6 mice	CUMS	Ber	i.g	2.5, 5, and 10 mg/kg, for 1 week	OFT, FST, NSFT	Ber reduced the immobility in the FST, increased the proportion of distance in the center, the total walking distance, total walking time, and central activity time in the OFT, and improved the latency period in the NSFT.
Gong et al. (2019)	C57BL/6 mice	CORT intervention	Ber	i.g	150 mg/kg, for 14 days	SPT	Ber increased the sucrose intake in the SPT.

Ber, berberine; GRb1, ginsenoside Rb1; BBH, berberine hydrochloride; HP-β-CD, hydroxylpropyl-β-cyclodextrin; EVO, evodiamine; CUMS, chronic unpredictable mild stress; CRS, chronic restraint stress; CORT, corticosterone; OFT, open-field test; FST, forced swimming test; TST, tail suspension test; EPM, elevated plus maze; SPT, sucrose preference test; NSFT, novelty-suppressed feeding test; ICR, institute of cancer research; SD, sprague dawley.

## 2 Potential mechanisms underlying the antidepressant effects of berberine

### 2.1 Berberine modulates neurotransmitter levels

The discovery of tricyclic antidepressants (TCAs) represented by promethazine, led to the formation of the monoamine theory of depression (Barsa and Kline, 1957) which proposed that depression may be caused by a decrease in the availability of monoamine neurotransmitters such as serotonin (5-HT) and noradrenaline (NE) in the central nervous system (CNS), and was one of the early hypotheses aiming to explain the pathophysiology of depression (Krishnan and Nestler, 2008). In the 1950s, the antitubercular drug, iproniazid, was shown to have antidepressant effects in tuberculosis patients, followed by the discovery that iproniazid inhibits the monoamine oxidase (MAO), which is involved in the catabolism of 5-HT, NE, and dopamine (DA) (Shulman et al., 2013). Meanwhile, two other pieces of evidence provide support for this theory, one of which is reserpine is thought to provoke depression essentially through catecholamine depletion (Strawbridge et al., 2023), and the other is that serotonin transporter knockout mice showed depression-like behaviors (Haenisch and Bönisch, 2011). What’s more, the first-line antidepressants increase acute delivery of monoamine neurotransmitters through inhibition of neuronal reuptake (e.g., SSRIs) or inhibition of degradation (e.g., MAOIs), which indicates the crucial role of monoamine neurotransmitters in the onset of depression. Although these hypotheses are constantly being updated, the strong link between monoamine neurotransmitters and the development of depression has never been questioned.

Ber can alleviate depression-like symptoms by modulating the levels of these neurotransmitters. Studies have shown that Ber could improve depressive-like behavior in mice by increasing the levels of NE, 5-HT, and DA in the hippocampus and frontal cortex as detected by high-performance liquid chromatography (HPLC) (Peng et al., 2007; Xu et al., 2021) and enzyme-linked immunosorbent assay (ELISA) (Huang et al., 2023). The 5-HT transporter (5-HTT) is an integral membrane protein that functions as a transporter protein and mediates the reuptake of 5-HT from inter-synaptic space, ensuring its recirculation into new cytoplasmic vesicles, and thus the duration and intensity of the biological action of 5-HT is largely dependent on 5-HTT (Iurescia et al., 2016). A study in the immortalized rat raphe-derived neuronal cell line RN46A cells showed that Ber (100 μM) can increase the mRNA and protein expression of 5-HTT, thereby enhancing the reuptake of 5-HT, which mechanism similar to that of SSRIs (Hu et al., 2012). Furthermore, Ber can also increase 5-HT levels in the hippocampus by regulating enzymes such as tryptophan 5-hydroxylase-1 (TPH1) and indoleamine 2,3-dioxygenase-1 (IDO1), thus shifting the kynurenine (KYN) pathway in tryptophan metabolism more towards the 5-HT pathway for the treatment of depression (Wang et al., 2022c; Ge et al., 2023). In addition, Ber may be an agonist of tyrosine hydroxylase (TH) in *Enterococcus*, which could lead to the production of L-dopa by the gut microbiota and finally transform into DA in the brain through a vitamin-like effect, thereby improving the brain function (Wang et al., 2021). We hypothesize that berberine may treat depression by modulating gut microbiota, which are likely important players in the diagnosis and treatment of depression due to their involvement in the bidirectional communication system of the gastrointestinal tract with the brain (Cryan et al., 2019).

### 2.2 Berberine enhances hippocampal neurogenesis

The cause of depression is far from being a simple deficiency of central monoamines. Subsets of depressed patients have been observed to exhibit volumetric reductions within the hippocampus and other forebrain regions, providing support for another prevalent hypothesis regarding depression, which posits a crucial role for neurodevelopmentally expressed growth factors in regulating plasticity within the adult brain (Monteggia et al., 2004). Several antidepressant treatments such as SSRIs, MAOIs, and SNRIs exhibit a notable cellular effect in the induction of adult hippocampal neurogenesis, a process characterized by the mitotic division of neural progenitors residing in the subgranular zone (SGZ) of the hippocampal region, leading to the formation of new neurons that subsequently undergo differentiation and integration within the dentate gyrus (DG) (Pittenger and Duman, 2008). Also, antidepressants could elevate the levels of various growth factors within the hippocampus, potentially through the modulation of cyclic adenosine monophosphate (CREB) or other transcription regulators, which exerts a significant influence on the process of neurogenesis (Krishnan and Nestler, 2008), further indicating the importance of neurogenesis in combating depression.

Ber could protect hippocampal nerves directly. MicroRNA (miR), a non-coding RNA with a size of approximately 22 nucleotides, frequently modulates gene expression at the post-transcriptional level. Mounting evidence indicates numerous miRNAs are specifically expressed or enriched in the brain, with aberrant miRNA expressions accompanying various neurological disorders in depression sufferers as well as depressive-like animals (Allen and Dwivedi, 2020; Fan et al., 2022). Studies showed that miR-34a overexpression in depressed mice impaired neurogenesis, and targeted inhibition of miR-34a expression by Ber could reverse this process and play an antidepressant role (Yi et al., 2021). Previous research suggested disrupting Jun N-terminal kinase (JNK)-Akt signaling could prevent hippocampal neuron apoptosis during ischemic brain damage (Gong et al., 2016). Zhang et al. demonstrated that the insulin-like growth factor receptor (IGFR) inhibitor remarkably enhances JNK and Akt expression, thereby inhibiting the Ber-augmented proliferation in hippocampal pyramidal neurons, which indicated a potential neuroprotective role for Ber (2 mg/kg/d) in the facial nerve axotomy damage mice model (Zhang et al., 2018).

In addition, Ber can also indirectly promote neurogenesis by modulating the levels of brain-derived neurotrophic factor (BDNF). BDNF, a neurotrophic factor that increases the proportion of neural stem cells that differentiate into neurons, and promotes the survival, proliferation, and maturation of neurons in the adult olfactory bulb and DG (Eliwa et al., 2017), has been validated as a key factor in promoting synaptic plasticity for antidepressant effects (Erickson et al., 2012; Zhang et al., 2016). Wang et al. demonstrated that exogenous BDNF administration and genetically engineered deletion of the DG resulted in the induction and attenuation of antidepressant response, respectively (Wang et al., 2022). Ber (100 mg/kg) can attenuate the depressive-like behavior (detected by SPT, FST, and open-field test (OFT)) by increasing BDNF expression in the hippocampal CA1 region (MA et al., 2012; Shen et al., 2016), and overexpressing BDNF can reverse the effects of miR-34b-5p and miR-470-5p on depressive-like behavior in CUMS mice (Zhan et al., 2021). Moreover, in de-ovulated model mice, the detection of CREB and eukaryotic translation elongation factor 2 (eEF2) suggested that Ber (10 mg/kg) can improve depression through the BDNF/CREB/eEF2 pathway, and the onset of action is 2–4 weeks faster than SSRI (Fan et al., 2017).

### 2.3 Berberine improves HPA axis function

The HPA axis is an important component of the neuroendocrine system, which has a close relationship with depression, with up to 40%–60% of depressed patients having hypercortisolemia or other HPA axis abnormalities (Keller et al., 2017). The activation of the HPA axis is characterized by an increase in hypothalamic production of corticotrophin-releasing factor (CRF), followed by increased pituitary release of adrenocorticotrophin (ACTH). There is strong evidence that stressful situations activate the HPA axis and increase circulating levels of glucocorticoid (GC) (Haleem and Gul, 2020), while overactivity of the HPA axis and increased circulating GC can affect brain serotonin and dependent responses to stress, precipitating depression (Haleem, 2022). Moreover, the observed correlation between heightened cortisol levels and the onset of depression may be attributed to the deleterious impact of excessive adrenal activity on the hippocampus (Mikulska et al., 2021). Chronic stress or the dysregulation of GC negative feedback receptors can result in elevated GC levels that can lead to significant damage to the hippocampus and hypothalamus (Hu et al., 2016). Ultimately, this neurogenic damage can lead to the proliferation of oligodendrocytes and exacerbation of depressive symptoms (Komoltsev and Gulyaeva, 2022). Chronic antidepressant treatment can restore the negative feedback function of the HPA axis, which either precedes or coincides with the relief of depression symptoms (Gobinath et al., 2014).

At present, many studies have demonstrated that Ber inhibits the abnormal activity of the HPA axis. In chronic unpredictable mild stress (CUMS)-induced mice, Ber (150 mg/kg) combined with ginsenosides could upregulate the expression levels of BDNF and downregulate the levels of corticosterone (CORT) and ACTH in plasma. Thereby attenuating depressive-like behaviors, including reducing the immobility time in FST, upregulating the percentages of sugar water preference in SPT and the activity times in EPM, increasing the maximum travel distance, total travel distance, and time spent at the area center in the OFT (Zhang et al., 2021). For the upstream hormones of CORT and ACTH, Ber (50 mg/kg) significantly reduced the expression of hypothalamic CRH and TH and showed greater improvement in depression and anxiety-like behavior (detected FST, EPM) in chronic morphine withdrawal rats (Lee et al., 2012). In addition, since excessive CORT is one of the important triggers for the onset of depression, quantitative proteomics of depressed mice revealed the inhibitory effects of CORT on the expression of mitochondrial oxidative phosphorylation-related proteins, and Ber could antagonize this effect and protect the neuronal physiological functions, which might be one of the mechanisms of the antidepressant effects of Ber (Gong et al., 2019).

### 2.4 Berberine reduces oxidative stress

Oxidative stress is another important factor that impairs neuroplasticity and contributes to the development of depression, it serves as a primary catalyst for neurodegeneration because reactive oxygen species (ROS) possess a profound relationship with a diverse array of pathophysiological processes (Bitanihirwe and Woo, 2011). When cells fail to maintain redox homeostasis and consequently generate proinflammatory mediators, cell necrosis ensues. The brain is particularly vulnerable to oxidative stress due to its elevated oxygen consumption, substantial lipid content, and relatively weak antioxidant defense system (Fesharaki-Zadeh, 2022). A clinical investigation demonstrated an elevated level of serum malondialdehyde (MDA) among individuals suffering from MDD, in comparison to a control population (Sarandol et al., 2007). In CUMS-induced depressed mice, the synthesis of peroxides such as MDA increased and the activity of antioxidant enzymes such as superoxide dismutase (SOD) and glutathione peroxidase (GSH-Px) decreased (Cheng et al., 2018), and antidepressant drugs could increase the levels of antioxidant enzymes, including catalase (CAT), SOD, and GSH-Px, in depressed patients or animals (Sherkawy et al., 2018; Meejuru et al., 2021; Mishra et al., 2021), the above suggests that improving the oxidative stress state might be an important direction for the treatment of depression.

In the study for type 2 diabetes mellitus (T2DM) model mice, Ber could increase the mRNA expression of SOD in the liver and the activities of SOD and CAT in the kidney tissue (Chatuphonprasert et al., 2013), which showed that Ber has a role in combating oxidative stress. What’s more, Ber was involved in the regulation of the GSH/GSH-Px antioxidant system in diabetic patients (Ma et al., 2018), indicating that Ber plays a role in ameliorating oxidative stress, and studies have shown that many signaling pathways may be involved. Sirtuin1 (SIRT1) is a deacetylase with excellent antioxidant properties whose expression level is significantly increased by Ber and triggers the transcription of forkhead box protein O (FoxO) target genes, including SOD, that affects the oxidative stress state (Hill et al., 2000; Chen and Yang, 2017). Ber mediates the inhibition of oxidative stress through the nuclear factor erythroid 2-related factor 2 (Nrf2) pathway (Yang et al., 2011; Mo et al., 2014) and the antioxidant activity of Ber can be eliminated by pharmacological blockade of Nrf2 in neurons and macrophages, verifying that the effects of Ber may be related to Nrf2 (Wen et al., 2020). However, fewer studies are validating the antidepressant effects of Ber from the perspective of oxidative stress, and the association between the two needs to be further explored in the future.

### 2.5 Berberine can inhibit inflammatory responses

Oxidative stress is always linked to inflammation. Inflammatory cells produce ROS, which can activate intracellular signaling and lead to the activation of proinflammatory genes. Peripheral cytokines undoubtedly have a role in behavioral effects, as evidenced by data indicating that blocking peripheral cytokines tightens the blood-brain barrier (BBB), and stopping BBB breakdown demonstrates antidepressant effects (Cheng et al., 2018). Beurel et al. demonstrated that peripheral cytokines can reach the brain maybe through “leaky” regions of the BBB, through a neural route via afferent nerve fiber cytokine receptors that relay the signal to the brain parenchyma, and through infiltration of immune cells (Beurel et al., 2020). At the molecular level, proinflammatory cytokines can reduce the supply of 5-HT, DA, and NE by increasing the expression and function of presynaptic 5-HT reuptake transporter proteins and activating the IDO to reduce related monoamine precursors (Maes et al., 2011). Moreover, inflammation affects growth factors, such as BDNF in the DG of the hippocampus, resulting in the damage of neuronal integrity, including neurogenesis, long-duration potentiation, and dendritic germination (Miller and Raison, 2016), which is important in the onset of depression. A meta-analysis found that depression is associated with concurrent and future inflammation in children and adolescents (Colasanto et al., 2020), Similarly, depressed patients were confirmed to have greater levels of proinflammatory cytokines such as tumor necrosis factor-α (TNF-α), interleukin-6 (IL-6), IL-1, IL-4, IL-5, IL-12, interferon-γ (IFN-γ), and C-reactive protein (CRP) in their blood (Hussain et al., 2016), while antidepressant treatment significantly could reduce peripheral levels of IL-6, TNF, IL-10 (Köhler et al., 2018). Furthermore, anti-inflammatory drug supplementary use of antidepressants appears to boost antidepressant efficacy, and treatment-resistant depressive patients may benefit from anti-inflammatory drugs as well (Raison et al., 2013). The above supports that inflammation is closely associated with the pathogenesis of depression.

The anti-inflammatory effects of Ber have long been well documented (Zhang et al., 2019; Wang et al., 2020; Naz et al., 2022), and many studies have shown that the effects of Ber in reducing neuropsychiatric symptoms are related to its anti-inflammatory effects. Ber (100 mg/kg) reduced the levels of IL-1β, IL-6, and TNF-α in the hippocampus and inhibited the activation of microglia in mice, thus alleviating their depressive symptoms (detected by SPT, novelty-suppressed feeding test (NSFT)) induced by CUMS (Liu et al., 2017). In another animal model, depressive symptoms complicated by inflammatory pain were significantly improved after Ber (50 mg/kg) intervention, which may be related to the reduction of IL-1β, IL-6, and TNF-α levels (Xu et al., 2018). Meanwhile, proteomics analysis of reserpine-induced depressed mice revealed that retinoic acid-inducible gene I (RIG-I) was highly expressed in the model group while negative in the Ber group, whereas RIG-I-mediated neuroinflammation may be involved in the pathogenesis of depression (Yang et al., 2022). In addition, activation of neuronal nitric oxide synthases (NOS) raises the concentration of NO, which ultimately leads to the development of depression (Adell, 2020; Kang et al., 2020), while the inhibitory effects of Ber on inducible NOS has been recognized (Zhu et al., 2018; Zhu et al., 2019). The tripartite motif (TRIM) family is a subfamily of E3 ubiquitin ligases that regulate the ubiquitination of target proteins in biological processes such as proliferation, apoptosis, development, differentiation, inflammation, and immunology. Yang et al., 2023 showed that Ber inhibits NLRP3 inflammasome activity by increasing Trim65 conjugation to NLRP3 and NLRP3 ubiquitination, effectively alleviates depressive symptoms (detected by SPT, FST, OFT, and tail suspension test (TST)), and reduces hippocampal neuronal functional damage in CUMS mice.

## 3 The effect of Chinese medicine prescription containing berberine on depression

Traditional herbal formulas tend to target more than a single herb and therefore have a more multifaceted therapeutic effect. Ber is one of the most important components in *Rhizoma Coptidis* (Huang Lian, HL), and current research has found that many formulas containing HL have antidepressant effects. Table 2 presents relevant information on the related studies.

**TABLE 2:** The *in vivo* studies on the antidepressant effects of traditional herbal formulas which containing berberine.

Formulas	Components	Ref.	Animals	Modeling methods	Usage	Dose and duration	Evaluation methods	Functions
Jiao-tai-wan (JTW)	*Rhizoma Coptidis* and *Cinnamon*	Bai et al. (2022)	Kunming mice	CORT intervention	i.g	2.1 and 4.2 g/kg, for 2 weeks	OFT, EPM, FST, TST	JTW increased the travel distance and time spent in the center in the OFT, improved the numbers into open arms and time on open arms in the EPM, and reduced the swing immobility time and floating immobility time in the TST and FST.
		Tang et al. (2022)	C57BL/6 mice	CRS	i.g	1.6 and 3.2 g/kg, for 21 days	OFT, TST, FST	JTW (3.2 g/kg) decreased the immobility time in the TST and FST and increased the total distance traveled in the OFT.
		Jiao et al. (2021)	SD rats	CUMS	i.g	0.75, 1.5, and 3 g/kg, for 14 days	OFT, SPT	JTW improved the sucrose preference in the SPT, and 3 g/kg JTW increased the upright numbers and crossing numbers in the OFT.
		Zhe et al. (2017)	ICR mice	LPS intervention	i.g	4.2 and 8.4 g/kg, for 7 days	FST, SPT, OFT, TST	JTW increased the sucrose preference in the SPT, decreased the immobility time in the TST and FST, and improved the crossings, rearing, and grooming numbers in the OFT.
Zuojin pill (ZJP)	*Rhizoma Coptidis* and *Evodia rutaecarpa*	Tao et al. (2023)	C57BL/6 mice	CUMS	i.g	450 and 910 mg/kg, for 3 weeks	SPT, FST, TST, OFT	ZJP increased the percent sucrose preference in the SPT and decreased the immobility time of the TST and FST, while having no significant effect on central movement distance in the OFT.
		Wang et al. (2023)	C57BL/6 mice	CUMS	i.g	225, 450, and 910 mg/kg, for 3 weeks	TST, SPT, FST	ZJP increased the sucrose preference level in the SPT and decreased the duration of immobility in the TST and FST.
		Wang et al. (2020)	SD rats	CUMS	i.g	0.6 and 1.2 g/kg, for 5 weeks	OFT, SPT	ZJP improved the sucrose preference in the SPT, ZJP (1.2 g/kg) increased the crossing, grooming, and rearing, and decreased time spent in the central area in the OFT.
		Wang et al. (2013)	ICR mice	reserpine intervention	p.o	5, 10, and 20 mg/kg, for 10 days	TST, FST, OFT	The ethanol extract of ZJP decreased the immobility time in the FST at 5 and 10 mg/kg, reduced the immobility time in the TST at 5 and 20 mg/kg, and did not affect the crossings and rearings in the OFT.
HuangLian JieDu Decoction (HLJDD)	*Rhizoma Coptidis, Radix Scutellariae, Cortex Phellodendri,* and *Fructus Gardeniae*	Qu et al. (2021)	C57BL/6 mice	CUMS	p.o	590 mg/kg, for 2 weeks	FST, OFT, NSFT	HLJDD decreased the immobility time in the FST, reduced the latency to feed in the NSFT, and increased total traveling time and distance, distance traveled at the center, and travel duration in the OFT.
		Zheng et al. (2023)	C57BL/6 mice	DSS intervention	p.o	2 and 4 g/kg, for 7 weeks	OFT, EPM, NORT	HLJDD increased the central travel distance and time ratio in the OFT, increased the time spent exploring the novel object in the NORT, and improved the time and distance on exploring the open arms in the EPM.
Banxia Xiexin Decoction (BXXXD)	*Pinelliae Rhizoma, Scutellariae Radix, Zingiberis Rhizoma, Ginseng Radix, Glycyrrhizae Radix, Coptidis Rhizoma,* and *Jujubae Fructus*	Liao et al. (2023)	C57BL/6 mice	High fat combined with bind stimulation	i.g	0.45, 1.35, and 4.05 g/kg, for 16 weeks	SPT, OFT, TST	BXXXD (1.35 and 4.05 g/kg) improved the rate of sugar-water consumption in the SPT, decreased the total motor distance and the central residence time in the OFT and the immobility time in the TST.

CUMS, chronic unpredictable mild stress; CRS, chronic restraint stress; CORT, corticosterone; LPS, lipopolysaccharide; DSS, dextran sulfate sodium; OFT, open-field test; FST, forced swimming test; TST, tail suspension test; EPM, elevated plus maze; SPT, sucrose preference test; NSFT, novelty-suppressed feeding test; NORT, novel object recognition test; ICR, institute of cancer research; SD, sprague dawley.

### 3.1 Jiao-tai-wan

**FIGURE 2:**
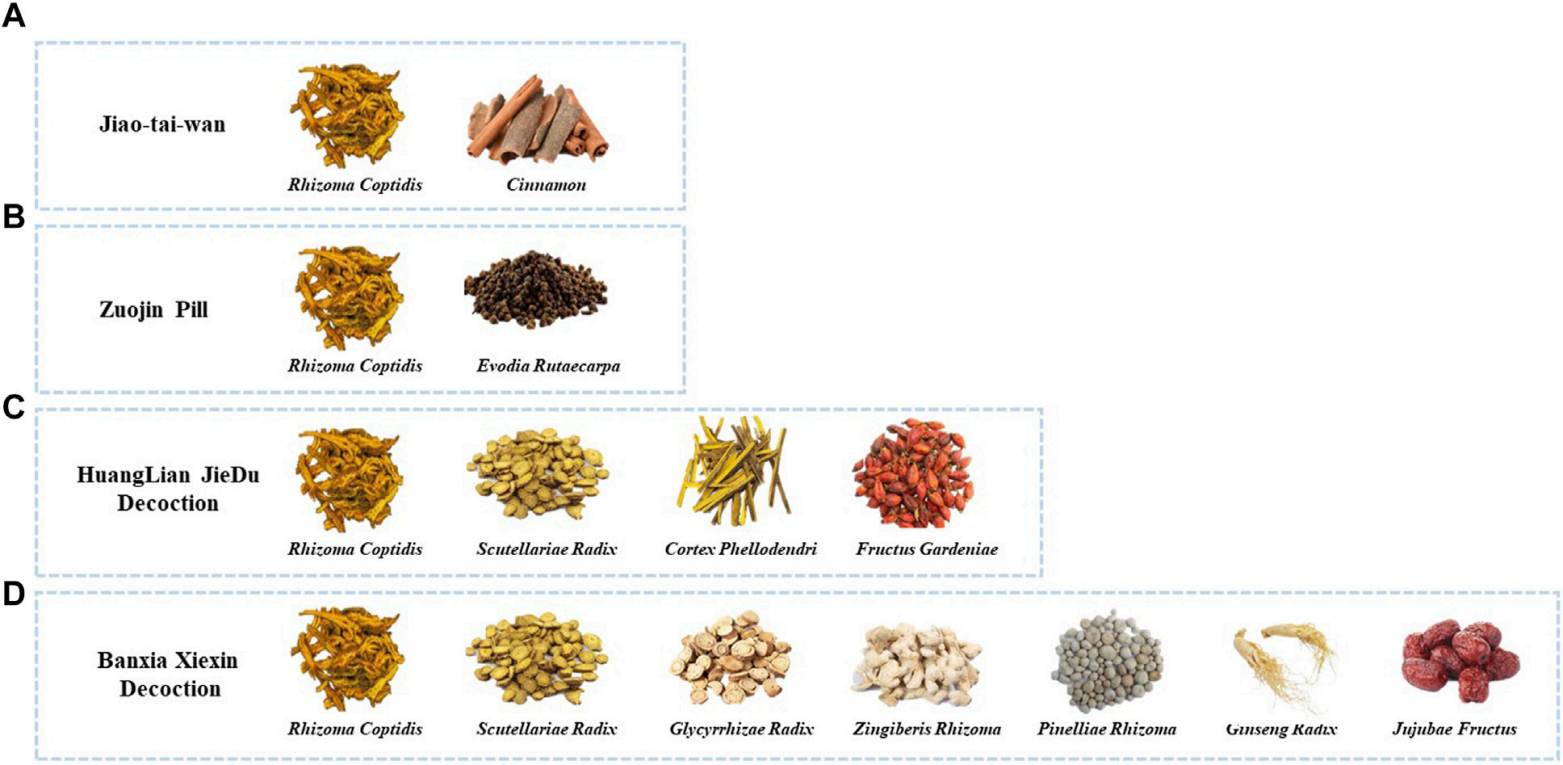
The components of the traditional herbal formulas in this review. **(A)** Chinese herbal medicine contained in Jiao-tai-wan. **(B)** Chinese herbal medicine contained in Zuojin Pill. **(C)** Chinese herbal medicine contained in HuangLian JieDu Decoction. **(D)** Chinese herbal medicine contained in Banxia Xiexin Decoction.

JTW, composed of *Rhizoma Coptidis* and *Cinnamon* (Figure 2A), has been applied for insomnia since ancient times, and its antidepressant effects are been explored. JTW can ameliorate depression-like symptoms in depression mice induced by chronic restraint stress (CRS), and has a protective effect on the damage to hippocampal neurons (Tang et al., 2022). Bai et al., 2022 found that JTW could ameliorate CORT-induced depressive-like behaviors and neuronal damage and enhance the levels of monoamine neurotransmitters in the serum of mice, which were also seen in the LPS-induced mice (Zhe et al., 2017). The therapeutic effects of JTW in the above experiments all involved an anti-inflammatory response. In addition, the metabolomics of serum from CUMS-induced rats showed that the antidepressant effects of JTW may be attributed to the regulation of amino acid metabolism, glycerophospholipid metabolism, and energy metabolism (Jiao et al., 2021).

### 3.2 Zuojin pill

ZJP, a classic herbal formula consisting of *Rhizoma Coptidis* and *Evodia Rutaecarpa* (Figure 2B) is widely used clinically to treat gastrointestinal diseases, and there have been confirmed that ZJP may have a role in alleviating depressive-like behavior. Wang et al. found that ZJP can improve CUMS-induced depression-like behavior via the TPH2/5-HT pathway (Wang et al., 2023). And the anti-inflammatory and antidepressant effects of ZJP are primarily attributed to the promotion of the ubiquitination of MyD88 and the inhibition of the activation of downstream inflammatory signals (Wang et al., 2020; Tao et al., 2023). Except for ZJP itself, the ethanol extract of ZJP also showed antidepressant-like effects in reserpine-induced depressed mice with a mechanism involving the central monoaminergic neurotransmitter system (Wang et al., 2013).

### 3.3 Huanglian Jiedu decoction

HLJDD includes *Rhizoma Coptidis*, *Scutellariae Radix*, *Cortex Phellodendri*, and *Fructus Gardeniae* (Figure 2C), and has been implicated as effective in treating inflammation-related diseases. HLJDD was able to alleviate depressive-like behaviors in colitis mice by inhibiting the Trem2/Dap12 signaling pathway in the microglia of the lateral habenula (Zheng et al., 2023). Additionally, network pharmacology analysis and metabolomics examination revealed that tryptophan metabolism serves as the primary target for HLJDD in CUMS mice, and SLC6A4 and MAOA within the tryptophan metabolic pathway were effectively modulated by Ber, baicalein, tetrahydro berberine, candicine, could be classified as the primary antidepressant targets for HLJDD (Qu et al., 2021), highlighting the key role of Ber in HLJDD.

### 3.4 Banxia Xiexin decoction

BXXXD is a formula consisting of seven herbs including *Pinelliae Rhizoma*, *Scutellariae Radix*, *Zingiberis Rhizoma*, *Ginseng Radix*, *Glycyrrhizae Radix*, *Coptidis Rhizoma*, and *Jujubae Fructus* (Figure 2D), which can lower lipids and alleviate depressive disorders, but fewer studies have been conducted to date. Liao et al. showed that BXXXD may exert a therapeutic effect by modulating the abundance of gut microbiota and thus intervening lipid metabolism in the peripheral and hippocampus (Liao et al., 2023). Another network pharmacology suggested the antidepressant effects of BXXXD are related to drug response, steroid metabolism, lipid metabolism, inflammatory response, oxidative stress response, and other biological functions (Yu et al., 2020), which need further validation.

## 4 Discussion

**FIGURE 3:**
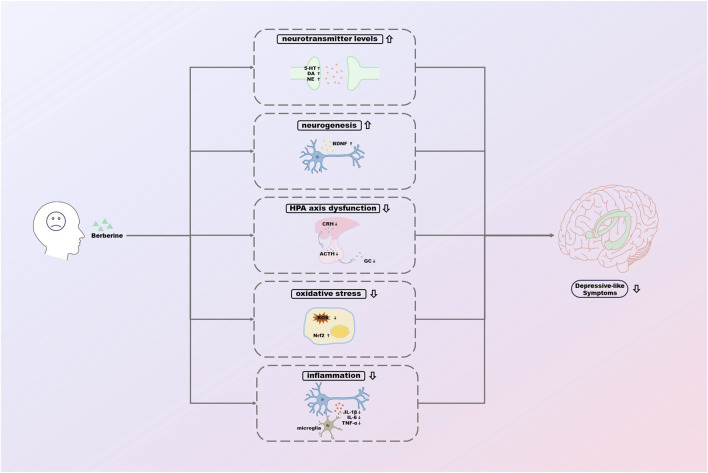
The potential mechanisms of antidepressant effects of berberine.

This paper reviews the potential mechanisms of antidepressant effects of Ber based on the existing studies (Figure 3). However, there is still a long way to go before Ber can truly be used as an antidepressant in the clinic.

One problem that cannot be avoided by applying Ber in the clinic is its low bioavailability. As an alkaloid with poor aqueous solubility (Shen et al., 2016), the bioavailability of Ber is less than 1% of the dose in rats, mostly owing to high intestinal first-pass metabolism (more than 98% of the absorbed amount), insufficient intestinal absorption (about 50% of the dose), and hepatic first-pass metabolism (20%–30% of that reached portal vein) (Murakami et al., 2023). The intrinsic mechanism may be related to the fact that Ber is P-glycoprotein (P-gp) (Zhang Y. et al., 2019), and cytochrome P450s (CYPs) (Johnson et al., 2002) expressed abundantly in the small intestine and the liver. Given the above, it has been shown that the bioavailability can be effectively improved by changing the route of administration (Murakami et al., 2023), co-administration with absorption enhancers (Xu et al., 2021; Wang et al., 2022), formulations containing solubilizer exhibiting P-gp and/or CYPs inhibitors (Kwon et al., 2020), and development of Ber analogs or derivatives (Wang et al., 2020; Kohli et al., 2021).

In addition, there is a need to focus on the safety of Ber. Ber may be cardiotoxic (Zhi et al., 2015) and hepatotoxic (Moreira et al., 2022), and the LD50 (median lethal dosage) of intravenous and intraperitoneal berberine was 9.0386 mg/kg and 57.6103 mg/kg, respectively. Ber has a high oral safety dose, because the absorption of Ber by the animal’s intestine system has its limit, and no matter how much the orally administered dosage is raised, the absorption rate would not increase at this internal limit (Kheir et al., 2010). Table 3 compares the toxicity of Ber with some clinically used antidepressants. Of these antidepressants, ketamine seems particularly dangerous as a street drug. Intravenous ketamine and intranasal esketamine (in combination with antidepressants) have proven efficacy in the management of treatment-resistant depression and are thus of high interest (Reif et al., 2023), and they seem to exert effects within 1 day whereas antidepressants generally take weeks (Popova et al., 2019). However, after acute dosing, psychiatric, psychotomimetic, cardiovascular, neurological, and other side-effects were more commonly observed after ketamine treatment than after placebo in patients with depression (Short et al., 2018). Interestingly, one study showed Ber can inhibit avoidance memory impairment of Toxoplasma gondii-infected rat model of ketamine-induced schizophrenia (Gholizadeh et al., 2023). Meanwhile, *levo*-tetrahydropalmatine, one of tetrahydroprotoberberines, could increase the bioavailability of ketamine and promote the metabolism of ketamine (Du et al., 2020), so the combination of ketamine and Ber might be a valuable new idea.

**TABLE 3:** Comparison of toxicity between berberine and commonly used antidepressants.

Drugs	Toxicity	Drugs	Toxicity
Berberine	Cardiotoxicity	MAOIs	Serotonin syndrome
	Hepatotoxicity		Hypertensive crisis
NMDA antagonist (ketamine)	Sedation	Vilazodone	Drowsiness
	Dissociation		Vomiting
	Ulcerative or Interstitial Cystitis		Tachycardia
	Embryo-fetal Toxicity		Serotonin syndrome (altered mental status, autonomic instability, and neuromuscular abnormalities)
Trazodone	Arrhythmias	Mirtazapine	Disorientation
	Respiratory arrest		Drowsiness
	Coma		Impaired memory
	Priapism		Bradyarrhythmias
Bupropion	Tachycardia	Dextromethorphan/bupropion	Seizures
	Hypertension		Psychosis
	Seizure		Serotonin Syndrome
SSRIs	Drowsiness	TCAs	Dilated pupils
	Tremor		Absent bowel sounds
	QRS and QTc interval prolongation (especially with citalopram and escitalopram)		Constipation
	Potential serotonin syndrome (hyperthermia, hypertonia, hyperreflexia, clonus)		Urinary retention
			Electrocardiogram changes (tachycardia, hypotension, conduction abnormalities, QRS duration >100 msec)
			Sedation
			Seizures
SNRIs	Tachycardia		
	Hypertension		
	Electrocardiogram changes (e.g., prolongation of QT interval, bundle branch block, QRS prolongation), ventricular tachycardia		
	Changes in the level of consciousness (ranging from somnolence to coma)		
	Mydriasis		
	Serotonin syndrome		
	Rhabdomyolysis		
	Liver necrosis		
	Death		

MAOI, monoamine oxidase inhibitor; NMDA, n-methyl-d-aspartic acid; SSRI, selective serotonin reuptake inhibitor; TCAs, tricyclic antidepressants; SNRI, serotonin and noradrenalin reuptake inhibitors.

Clinical trials of Ber in the treatment of depression are still lacking. A clinical randomized controlled trial that included 164 patients showed that Ber hydrochloride improved performance on a depression scale in patients with irritable bowel syndrome (Chen et al., 2015). However, such a change might be related to the improvement of the patient’s intestinal symptoms and does not directly reflect the role of berberine in the treatment of depression. Another clinical study that included 52 opioid addicts demonstrated that there were no significant differences in depression, anxiety, stress, and sleep quality scores in the treatment group given capsules of *Berberis vulgaris* extract (Dabaghzadeh et al., 2023). Therefore, more rigorously designed and targeted clinical trials are needed to guide the clinical application of Ber in the future.

Due to the late discovery of the antidepressant effect of berberine, there are still some problems in the experimental research on this area: 1) Currently, the *in vitro* experiments of the antidepressant effects of Ber mainly used the HT22 cell line, and due to the less frequent use of primary cells and the lack of a recognized modeling method, the progress in the exploration of the mechanisms is slow. 2) The anti-oxidative stress effect of Ber is clear and there is a strong correlation between oxidative stress and depression, but there has not been a study directly focused on the relationship between the anti-oxidative stress and antidepressant effects of Ber, which needs to be explored in the future. 3) Doses (from 2 to 200 mg/kg) and duration (from 1 to 4 weeks) of Ber have varied considerably from study to study, and it has not yet been possible to determine an appropriate dose range for the treatment of depression. It is necessary to compare different doses and duration of Ber in different depression models to investigate the most reasonable dose, with the use of consistent behavioral tests.

In summary, the relevant studies suggest that the mechanisms of the antidepressant effects of Ber may be related to the regulation of neurotransmitter levels, enhancement of hippocampal neurogenesis, improvement of HPA axis function, reduction of oxidative stress, and inhibition of inflammatory responses. These pathways are essential in the pathogenesis of depression and are also crucial for the efficient treatment of depression. Ber, as a monomer of TCM with rich pharmacological effects, the exploration of its relevant mechanisms is still in its infancy, although there have been several studies on its antidepressant effects. We believe that the exploration of the efficacy of Ber will be deepened gradually, and the potential mechanisms of the antidepressant effects of Ber will be clarified, which will have a broader application prospect in the future.

## Figures and Tables

**Table:** 

**DALYs**	disability-adjusted life-years
**SSRIs**	selective serotonin reuptake inhibitors
**SNRIs**	serotonin and norepinephrine reuptake inhibitors
**MAOIs**	monoamine oxidase inhibitors
**MDD**	major depressive disorder
**TCM**	traditional Chinese medicine
**LPS**	lipopolysaccharide
**Ber**	berberine
**HPA**	hypothalamic-pituitary-adrenal
**CNKI**	Chinese National Knowledge Infrastructure
**TCAs**	tricyclic antidepressants
**5-HT**	serotonin
**NE**	noradrenaline
**CNS**	central nervous system
**MAO**	monoamine oxidase
**DA**	dopamine
**HPLC**	high-performance liquid chromatography
**ELISA**	enzyme-linked immunosorbent assay
**5-HTT**	5-HT transporter
**TH**	tyrosine hydroxylase
**TPH1**	tryptophan 5-hydroxylase-1
**IDO1**	indoleamine 2,3-dioxygenase-1
**KYN**	kynurenine
**SGZ**	subgranular zone
**DG**	dentate gyrus
**miR**	microRNA
**JNK**	Jun N-terminal kinase
**CREB**	cyclic adenosine monophosphate
**IGFR**	insulin-like growth factor receptor
**BDNF**	brain-derived neurotrophic factor
**OFT**	open-field test
**eEF2**	elongation factor 2
**CRF**	corticotrophin-releasing factor
**ACTH**	adrenocorticotrophin
**GC**	glucocorticoid
**CUMS**	chronic unpredictable mild stress
**CORT**	corticosterone
**FST**	forced swimming test
**SPT**	sucrose preference test
**EPM**	elevated plus maze
**ROS**	reactive oxygen species
**MDA**	malondialdehyde
**SOD**	superoxide dismutase
**GSH-Px**	glutathione peroxidase
**CAT**	catalase
**T2DM**	type 2 diabetes mellitus
**SIRT1**	sirtuin1
**FoxO**	forkhead box protein O
**Nrf2**	nuclear factor erythroid 2-related factor 2
**BBB**	blood-brain barrier
**TNF-α**	tumor necrosis factor-α
**IL-6**	interleukin-6
**IFN-γ**	interferon-γ
**CRP**	C-reactive protein
**RIG-I**	retinoic acid-inducible gene I
**NSFT**	novelty-suppressed feeding test
**NOS**	nitric oxide synthases
**TRIM**	tripartite motif
**TST**	tail suspension test
**HL**	Huang Lian
**JTW**	jiao-tai-wan
**CRS**	chronic restraint stress
**HLJDD**	HuangLian JieDu Decoction
**ZJP**	Zuojin pill
**BXXXD**	Banxia Xiexin Decoction
**P-gp**	P-glycoprotein
**CYPs**	cytochrome P450s
**LD 50**	median lethal dosage
